# Isolation of human satellite cells using CD29/CDH15 and a nanoparticle-based live Pax7 mRNA detection system, and their expansion with extracellular matrix and its derivatives

**DOI:** 10.1186/s13287-026-05134-x

**Published:** 2026-07-07

**Authors:** Shudong Zhao, Shubhankar Sood, Ruixiang Yu, Luis Arvizu Gutierrez, Takhmina Ayupova, Sara Taiyari, Jonathan Miles, Stephen Hamilton, Massimiliano Cerletti

**Affiliations:** 1 Division of Surgery and Interventional Science, Tissue Repair and Regeneration Centre, University College London, Royal Free Campus, London, NW3 2PF UK; 2 Centre for Surgical Innovation, Organ Repair and Transplantation (CSIORT), University College London, Royal Free Campus, London, NW3 2PF UK; 3 Centre for Bioengineering and Surgical Technology (BEST), University College London, Brockley Hill, Stanmore, HA7 4AP UK; 4 Orthopaedic Surgery, The Royal National Orthopaedic Hospital, Brockley Hill, Stanmore, HA7 4AP UK; 5 Breast and Plastic Surgery, The Royal Free Hospital, London, NW3 2QG UK

**Keywords:** Human satellite cells, Cell surface markers, Nanoparticle-based live mRNA detection probes, Skeletal muscle regeneration, ECM, Decellularization

## Abstract

Human skeletal muscle stem cells, also called satellite cells, have great potential for treating neuromuscular diseases and combating muscle aging. However, progress toward clinical use has been limited by the scarcity of donor tissue and the technical challenges of isolating, preserving, and culturing these rare cells. In this study, we introduce a new combination of cell surface markers and a gold nanoparticle-based live Pax7 mRNA detection system, both of which enable efficient isolation of human satellite cells. Additionally, we show that extracellular matrix (ECM) components support the maintenance of human satellite cells and improve their function in vivo. These findings provide integrated methods to enhance the production of human muscle-forming cells for translational and clinical applications.

**Supplementary Information:**

The online version contains supplementary material available at https://doi.org/10.1186/s13287-026-05134-x.

## Introduction

Human skeletal muscle stem cells, known as satellite cells, are crucial for muscle growth, maintenance, and repair throughout life [1]. Muscle regeneration highlights the dynamic interplay between intrinsic factors within satellite cells and extrinsic signals. Upon exposure to these cues, quiescent satellite cells re-enter the cell cycle, divide briefly to replenish the stem cell pool, or differentiate into the myogenic lineage, forming new muscle fibers [2, 3]. Satellite cells are identified by the transcription factor Pax7, which is expressed in both quiescent and proliferating cells during early commitment but not in later stages of differentiation [4].

As precursors to skeletal muscle, human satellite cells are essential for medical applications, including the treatment of neuromuscular diseases such as muscular dystrophy and age-related conditions. They serve as a source for therapeutic transplantation or as a reliable platform for drug testing [5]. The success of research and treatment depends primarily on a sufficient, pure supply of functional human satellite cells.

Satellite cells are a rare cell type found in human skeletal muscle [3]. Harvesting human muscle tissue is limited for ethical and clinical reasons. Because biopsies are invasive, muscle samples are typically small, ranging from 100 to 150 mg, especially in children [6]. The naturally low cell count, combined with limited donor tissue, yields very low amounts that are often too small for further use without extensive in vitro expansion. These challenges highlight the need to develop reliable methods for isolating and maintaining these cells to improve recovery and preserve the myogenic potential of human satellite cells for research and therapy.

Identifying and characterizing a cell population depends on specific biomarkers, including transcription factors and membrane proteins. Traditionally, intracellular markers have been preferred for identifying and analyzing cells based on their internal proteins and genetic signals [7]. However, this approach often requires fixing the cells, which prevents their use in subsequent tests. The secreted biomarker technique was initially developed to isolate immune cells, as these cells produce specific cytokines that can be traced back to their source [7]. Unfortunately, no similar method has been developed for satellite cells, making it difficult to label them based on the biomolecules they secrete. Recently, microRNA labeling techniques have gained popularity [8]; however, no commercially available antibodies specifically label human satellite cells using this method.

Currently, only a few methods have been reported for isolating human satellite cells. For example, the pre-plating technique separates cells by adhesive properties, thereby enriching satellite cells with lower adhesion [9]. This inexpensive and straightforward method, however, is time-consuming and often inefficient because of variable purity and the risk of fibroblast contamination. Additionally, the ice-cold treatment method has been used to enrich human satellite cells by selectively enhancing their survival under short-term hypothermic stress [10]. As a negative-selection approach, it removes non-satellite cells rather than positively labeling the target cells, resulting in inconsistent purity and strong dependence on the initial cellular composition of muscle biopsies. The use of cell surface markers remains the primary method for purifying these cells. However, the combination of surface markers for human satellite cells remains poorly understood, and no widely accepted or effective sorting strategy has been established. Therefore, current challenges in detecting and isolating human satellite cells highlight the need to develop more effective approaches to address this gap.

In this study, we present a new combination of specific cell surface markers and a gold nanoparticle-based live Pax7 mRNA detection system, both used to isolate human satellite cells, along with ECM components that support their maintenance. These advances could enhance the production of human muscle-forming cells, support stem cell therapies, and improve treatments for traumatic muscle injuries.

## Materials and methods

### Animals

C57BL/6 mice, 3–5 months old, and athymic nude Foxn1nu mice, 4–6 weeks old, were obtained from Charles River Laboratories (UK). Animal work was conducted in accordance with the ARRIVE guidelines 2.0. Throughout the experimental period, all procedures strictly adhered to humane care principles and ethical standards for animal research. All procedures were approved by the Institutional Animal Care and Use Committee.

### Human skeletal muscle biopsy

Discarded skeletal muscle samples (3–6 cm) weighing 150–1000 mg were collected from approximately 20 healthy individuals aged 40–56 years, in accordance with all relevant NHS ethical guidelines for consent. Each biopsy was placed in sterile PBS and processed immediately to prevent tissue degradation and cell death.

### Human satellite cell isolation

*Method 1a*: Single myofibers and myofiber-associated cells (MFAs) were isolated from rectus abdominis biopsies as described by Sherwood et al. [11], Cerletti et al. [12], and Cerletti et al. [13]. After isolation, myofiber-associated cells were stained with FACS antibodies to identify satellite cells: CD31^−^CD34^−^CD45^−^Mac1^−^β1-integrin^+^CDH15^+^. FACS data were collected using DIVA software (Becton & Dickinson (BD), FACS Aria III, Franklin Lakes). Antibodies used for flow cytometry included: CD31 Monoclonal Antibody (PE, Invitrogen, 1:200); CD34 Monoclonal Antibody (FITC, BD Pharmingen, 1:200); CD45 Monoclonal Antibody (PE, Invitrogen, 1:200); Mac1, CD11b Monoclonal Antibody (PE, Invitrogen, 1:200); β1-integrin, CD29 Antibody (APC/Cyanine7, BioLegend, 1:100); M-Cadherin/Cadherin-15, CDH15 Antibody (R&D Systems, 1:200); anti-sheep IgG biotin secondary antibody (Invitrogen, 1:1600); and APC Streptavidin (BioLegend, 1:200). Live cells were identified as calcein blue-positive (Invitrogen, 1:1000) and propidium iodide-negative (PI, 1 μg/mL, Sigma-Aldrich). Antibody incubations were performed in staining media (Hank’s Buffered Salt Solution (Gibco) with 2% donor fetal bovine serum (FBS, Thermo Fisher Scientific)) on ice for 20 min. *Method 1b*: Trimmed muscle biopsies were cultured in 6-well plates containing DMEM (4.5 g/L D-glucose, Gibco) with 20% FBS and 5 ng/ml bFGF (added daily; Sigma-Aldrich) for 7–10 days. Proliferating cells were stained for FACS to identify satellite cells, as described in Method 1a. *Method 2*: Myofiber-associated cells isolated as described above were stained for FACS to purify the CD31^−^CD34^−^CD45^−^Mac1^−^ cell population. Sorted CD31^−^CD34^−^CD45^−^Mac1^−^ cells were cultured and expanded in DMEM with 20% FBS at 37 °C until they reached 60–70% confluence, then incubated with the gold nanoparticle Pax7 mRNA probe.

### Nanoparticle-based probe system for real-time Pax7 mRNA detection and sorting

The nanoparticle-based live mRNA detection kit, which uses gold nanoparticles and includes Pax7-targeted, scrambled, and uptake control probes, was obtained from Merck Millipore (Pax7 Human-Cyanine 5 SmartFlare™ RNA Probe). Sixty to seventy percent confluent CD31^−^CD34^−^CD45^−^Mac1^−^ cells were incubated with the Pax7 probe at concentrations of 10–12 μL/mL for 24 or 36 h. Cy5 signals were detected by brightfield and fluorescence microscopy with excitation at 650 nm. CD31^−^CD34^−^CD45^−^Mac1^−^ cells positive for Cy5 were then sorted by FACS (Becton & Dickinson (BD), FACS Aria III, Franklin Lakes).

### Formation of multinucleated myotubes

Sorted human satellite cells were cultured in growth medium until reaching 70–80% confluence, then switched to differentiation medium (DMEM + 5% horse serum (HS, GIBCO)) for 7–10 days until elongated, multinucleated myotubes were visible.

### Immunofluorescence

Pax7 staining was performed on sorted cells from freshly prepared, cultured, and Pax7 nanoparticle-isolated samples. After fixation in 4% paraformaldehyde (PFA) for 15 min and permeabilization with 0.1% Triton X-100 (Sigma-Aldrich) for 15 min at room temperature, cells were incubated with 5% normal goat serum (NGS, Gibco) for 30 min. They were then incubated overnight at 4 °C with the Pax7 antibody (1:50, Developmental Studies Hybridoma Bank (DSHB)), followed by a secondary antibody (1:400, Alexa Fluor 488, Invitrogen) for 1 h. Myotubes were fixed and permeabilized as described above, treated with 5% NGS for 30 min, and stained with the MyHC antibody (1:200) for 2 h. Next, they were incubated with a secondary antibody (1:400, Alexa Fluor 594 or 488, Invitrogen) for 1 h at room temperature, followed by Hoechst 33342 staining (2 µg/mL, Invitrogen). Fluorescence images were captured using a Leica DMI4000 B inverted microscope (Leica).

### Extracellular matrix obtained from decellularized muscle (dECM)

Skeletal limb muscles (triceps, tibialis anterior (TA), gastrocnemius, quadriceps, and gluteus) from C57/BL6 mice were harvested and decellularized by incubating them in ultrapure water (30 mL per 1 g of muscle) with agitation for 2 days, with three water changes per day. This was followed by treatment with 1% (w/v) sodium dodecyl sulfate (SDS, Sigma-Aldrich) for 2 days, with one change per day, and with 1% (v/v) Triton X-100 (Sigma-Aldrich) for 30 min. The muscles were then washed with ultrapure water for at least 2 days, with daily water changes. After decellularization, the muscle tissue was lyophilized and ground into powder. Then, 100 mg of dECM was incubated with 10 mL of 0.1% (w/v) pepsin (Sigma-Aldrich) in 0.5 M acetic acid (Sigma-Aldrich) for 3 days with agitation. After digestion, the solution was allowed to settle and then filtered. The dECM hydrogel was formed by natural water evaporation, thereby preventing crosslinking-induced alterations to dECM protein structures that could affect protein function. The rheological behavior of dehydrated dECM gel and dECM coating solution was assessed using a rheometer (NETZSCH Kinexus) equipped with a parallel-plate geometry. All measurements were performed at 37 °C with a 0.3 mm gap. Strain sweeps (0.1–100% strain, 1 Hz) were conducted to identify the linear viscoelastic region, followed by frequency sweeps (0.1–10 Hz) at a constant strain of 0.1%. Storage modulus (G′), loss modulus (G″), and complex viscosity (η*) were recorded for comparison.

### Mass spectrometry and data analysis

Peptides from dECM were analyzed by liquid chromatography-mass spectrometry (LC–MS) on a Q Exactive mass spectrometer (Thermo Fisher Scientific, USA). Proteins were identified with MaxQuant and classified using the MatrisomeDB 2.0 database. The mass spectrometry proteomics data are available via ProteomeXchange under the identifier PXD073117.

### Human satellite cell expansion

48-well plates were pre-coated with 150 µl of dECM for at least 60 min, then seeded with twenty thousand human satellite cells. Cells were cultured for 5 or 10 days and analyzed for Pax7 expression. Similarly, ECM proteins were tested, including collagen IV (1 µg/cm^2^, BioTechne), laminin (1 µg/cm^2^, Gibco), nidogen (1 µg/cm^2^, BioTechne), perlecan (1 µg/cm^2^, BioTechne), and a mixture of these proteins at the same concentration. Twenty thousand human satellite cells were seeded onto uncoated or coated plates, cultured for 5 days, and then analyzed for Pax7 expression.

### Antibody blocking assay

Twenty thousand human satellite cells were suspended and preincubated with the anti-CD29 blocking antibody (10 µg/ml, Abcam) for 1 h at 37 °C before seeding. To maintain receptor blockade during culture, the blocking procedure was repeated on day 3 by splitting the cells. Satellite cells were analyzed for Pax7 expression on day 5, as described above.

### Human satellite cell transplantation

25,000 human satellite cells, treated with dECM or left untreated, were injected into the CDTX-injured tibialis anterior (TA) muscle (0.03 mg/mL, Sigma-Aldrich) of anesthetized (1–2% isoflurane, Sigma-Aldrich) athymic nude Foxn1nu mice (TA = 6 per group, n = 6) one day after injury. CDTX was injected into the TA without anesthesia. Mice were culled with CO_2_ (increasing concentration) and confirmed by cervical dislocation four weeks after transplantation. No predefined criteria were used to include or exclude mice, and no mice or data points were excluded from the analysis. TAs were harvested, processed, and examined in a blinded manner for human dystrophin, Pax7, and lamin A/C. Specifically, air-dried TA muscle sections were rinsed twice with PBS (2 min each) and blocked with the M.O.M. Mouse IgG Blocking Reagent (Vector Laboratories) for 1 h. After a brief incubation in M.O.M. Diluent (5 min, Vector Laboratories), the sections were incubated overnight at 4 °C with human dystrophin antibody (1:50; MANDYS102, DSHB), Pax7 (1:50, DSHB), or human lamin A/C (1:200, Abcam), all prepared in M.O.M. Diluent. This was followed by incubation with a secondary antibody (1:400, 1 h, Alexa Fluor 594 or 488, Invitrogen). The sections were then washed in PBS and mounted in mounting medium containing DAPI (Vector Laboratories). Fluorescence images were captured using a Leica DMI4000 B inverted microscope.

### ATPase staining

ATPase staining involved pre-incubating muscle sections in a pH 4.5 buffer for 2 min at 22–24 °C, followed by a brief water rinse. Sections were then incubated in ATP buffer at room temperature for 20–25 min, rinsed 2–3 times with 1% CaCl₂, stained with 1% Azure A for 1 min, and rinsed again. Preparations were dehydrated in ethanol at 50%, 70%, 95%, and 100%, cleared with xylene, and mounted with DPX (Sigma).

### Statistical analysis

Data were analyzed using GraphPad Prism (San Diego, USA) and reported as means ± standard deviation (SD). Normality was assessed using the Shapiro–Wilk test. Differences between two or more groups were evaluated using a t-test or analysis of variance (ANOVA) with Tukey HSD post hoc testing. A *p*-value less than 0.05 (*p* < 0.05) was considered statistically significant.

## Results

### A new set of cell surface markers for isolating human satellite cells

**Fig. 1:**
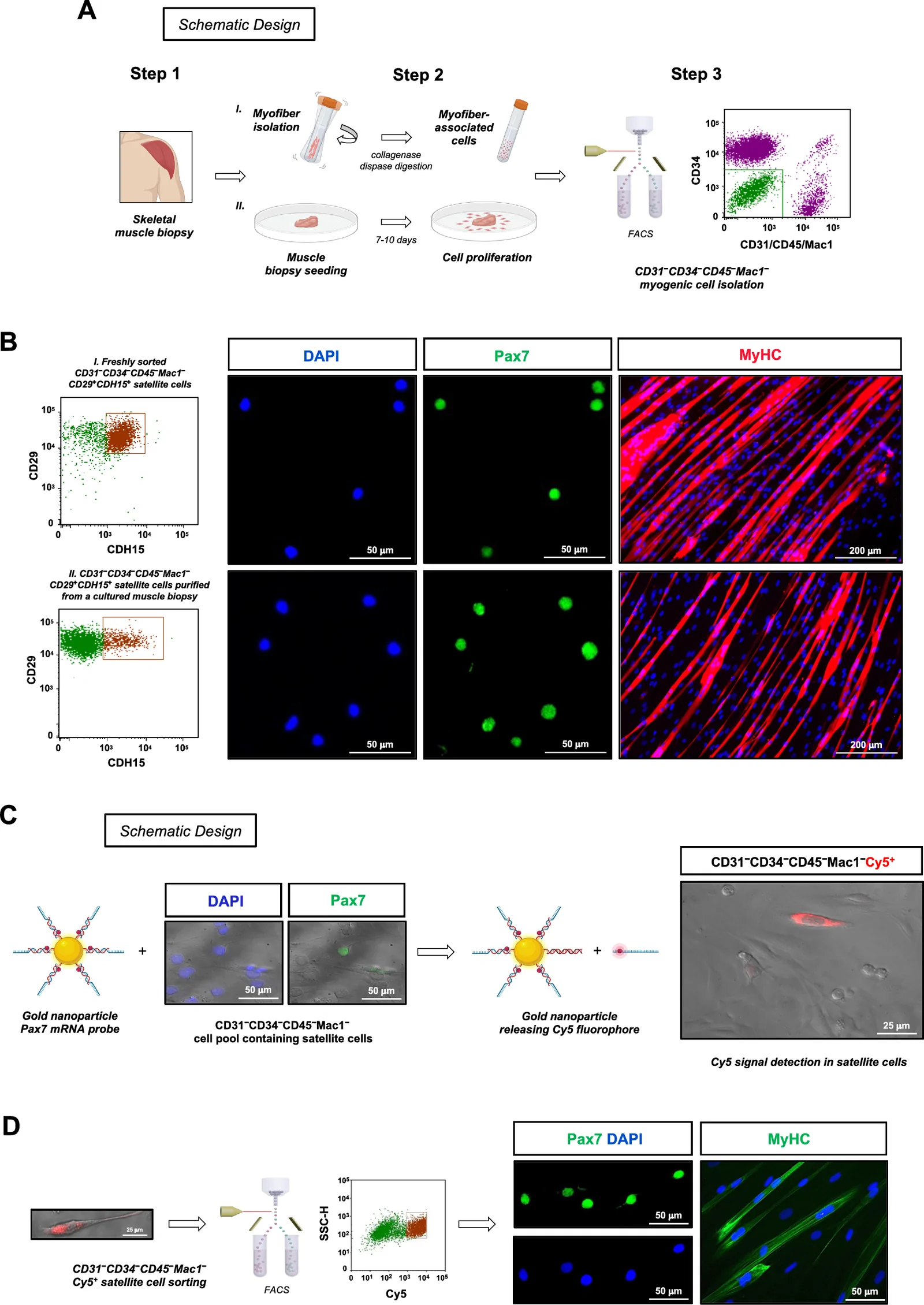
Purification of human satellite cells by positive selection for CD29 and CDH15 and by a gold nanoparticle system for live Pax7 mRNA detection. (**A**) Diagram of a processed or cultured muscle biopsy. The FACS plot shows exclusion of endothelial, stromal, and hematopoietic cells, thereby reducing non-myogenic contamination, and highlights the CD31^−^CD34^−^CD45^−^Mac1^−^ myogenic population (green). (**B**) Freshly sorted satellite cells (CD29^+^CDH15^+^, top FACS plot), previously gated for CD31^−^CD34^−^CD45^−^Mac1^−^, and satellite cells sorted from a cultured muscle biopsy (bottom FACS plot). Immunofluorescence images show purified Pax7^+^ satellite cells (green) and MyHC^+^ multinucleated myotubes (red) derived from differentiated satellite cells. Nuclei are stained blue. (**C**) Schematic of the nanoparticle-based live Pax7 mRNA detection system. Satellite cells are identified as alive (Cy5, red) or fixed (green) within the CD31^−^CD34^−^CD45^−^Mac1^−^ population. Nuclei are stained blue. (**D**) Immunofluorescence images of Cy5-sorted Pax7^+^ satellite cells (green) and derived MyHC^+^ multinucleated myotubes (green). Nuclei are stained blue. Scale bars: 25–200 μm

To isolate human satellite cells from a mixed myofiber cell population—whether from dissociated muscle samples or from migrating cells derived from cultured muscle biopsy explants—we used a cell surface marker-based approach. Human muscle biopsies were trimmed of fat and connective tissue and processed either by direct enzymatic digestion (as previously described [11–13]) or by trimming and culturing to promote cell migration. In both methods, cells associated with myofibers were stained with antibodies against CD31, CD34, CD45, and Mac1 to exclude endothelial, stromal, and hematopoietic lineages (Fig. 1A) [14, 15]. From the negative cell population (green), we performed positive selection for CD29 and CDH15. The percentage of satellite cells among total cells ranged from 0.3 to 0.6 in biopsies weighing 150–1000 mg, depending on sample quality and patient age. CD29 (β1-integrin) is crucial for anchoring satellite cells to the basal lamina and consistently correlates with the transcription factor Pax7 [16–18]. CDH15 (M-cadherin) is expressed in early myogenic progenitors and supports cell adhesion and extracellular matrix remodeling [19, 20]. Therefore, sorted CD31⁻CD34⁻CD45⁻Mac1⁻CD29⁺CDH15⁺ cells were tested for Pax7 expression, yielding a satellite cell population with over 98% purity, whereas sorting for CD29 alone captured only half of the satellite cell pool (Fig. 1B and data not shown). When cultured under 5% serum conditions, these cells differentiated robustly and efficiently formed MyHC⁺ multinucleated myotubes, confirming their myogenic potential in vitro (Fig. 1B). Our results demonstrate that combining CD29 and CDH15 effectively isolates functional human satellite cells from both fresh and cultured biopsy samples, underscoring this method's versatility for muscle research and regenerative medicine.

### Gold nanoparticle-based live Pax7 mRNA probe for detecting human satellite cells

Nanoparticle-based live mRNA probes effectively detect specific gene expression in living cells and have attracted growing interest in biomedical research [21]. Given the satellite cell-specific expression of Pax7, we used a gold nanoparticle-based live Pax7 mRNA detection system to assess its potential for isolating human satellite cells.

The nanoparticle-based live Pax7 mRNA probe uses an inert gold nanoparticle that is readily taken up by live cells through natural endocytic pathways. Multiple copies of a double-stranded Pax7 mRNA oligonucleotide are attached to the nanoparticle core. This oligonucleotide consists of two pre-hybridized strands: the capture strand, which specifically binds and detects Pax7 transcripts within cells, and the reporter strand, which carries a quenched Cy5 fluorophore. The live Pax7 mRNA detection probe was incubated with the CD31^−^CD34^−^CD45^−^Mac1^−^ cell pool enriched for satellite cells, allowing the probe to enter satellite cells and encounter the target Pax7 mRNA. The capture strand hybridized to Pax7 mRNA, displacing the nonspecifically bound reporter strand. As a result, the Cy5 fluorophore on the reporter strand was unquenched and detected using various fluorescence platforms (e.g., microscopes, FACS) (Figs. 1C and S1A, B). Pax7-expressing satellite cells were readily identified by their Cy5 red fluorescence, enabling visualization and real-time monitoring of cell behavior. The nanoparticle-based live Pax7 mRNA probe was validated by running it in parallel with scrambled and uptake controls (Fig. S1A, B). Using FACS, these transient Cy5^+^ cells were sorted with 96–97% Pax7 purity (Fig. 1D). Notably, sorted satellite cells showed typical myogenic potential, differentiating efficiently into MyHC^+^ multinucleated myotubes (Fig. 1D). Therefore, this gold nanoparticle-based live Pax7 mRNA detection method provides a promising approach for effective molecular tagging and single-cell mRNA quantification in live satellite cells, making it a useful tool for characterizing and isolating these cells.

The two human satellite cell isolation methods described above are not mutually exclusive; rather, they complement each other. Surface marker–based isolation offers a practical, scalable approach, whereas the nanoparticle-based live Pax7 mRNA probe enables detection and real-time tracking.

### The muscle extracellular matrix supports the growth and function of human satellite cells

**Fig. 2:**
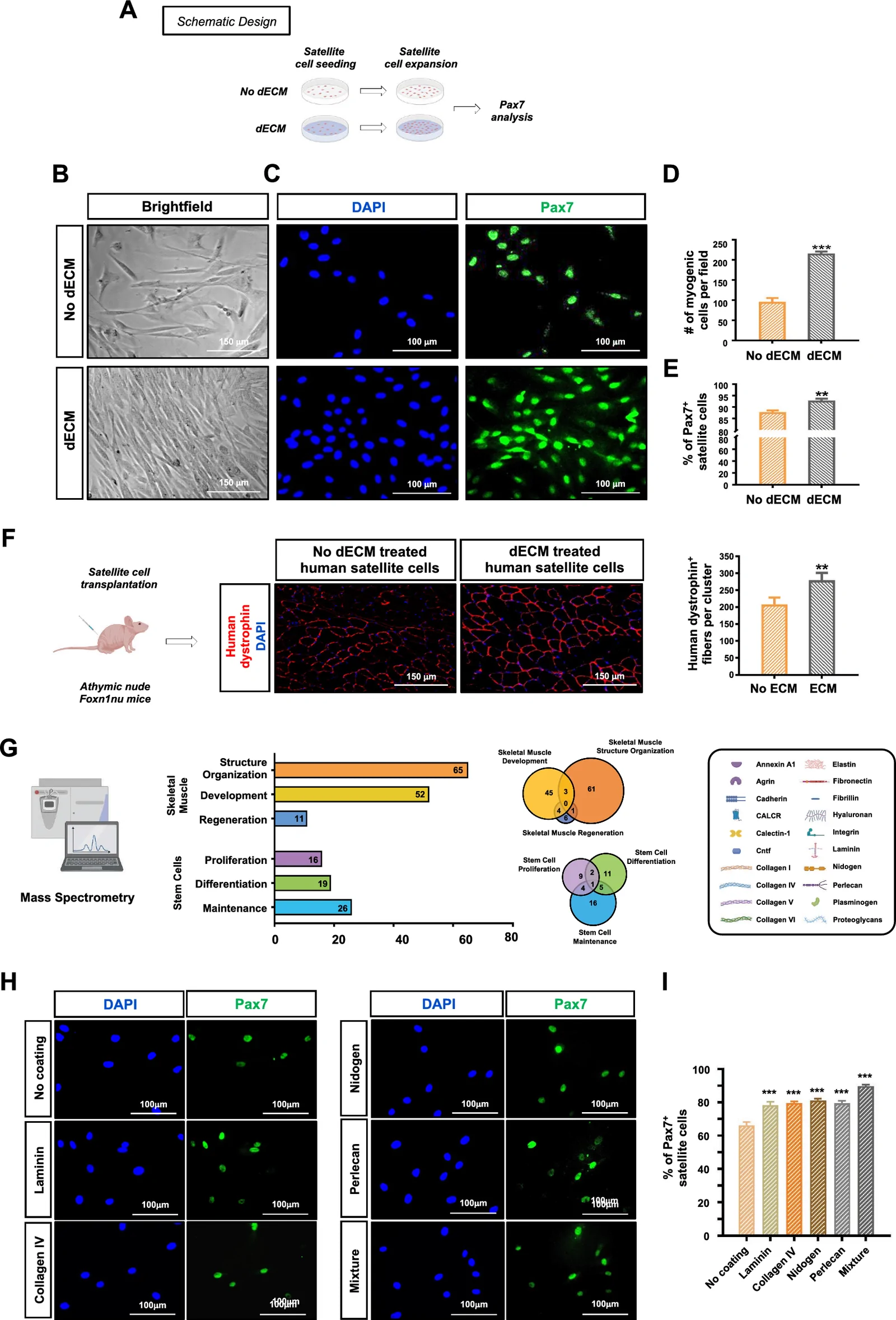
Mass spectrometry analysis of ECM from decellularized muscle identifies components that support human satellite cell maintenance. (**A**) Diagram illustrating human satellite cell seeding on dECM-treated plates and analysis of Pax7 expression. (**B**) Optical images and (**C**) immunofluorescence images of human satellite cells stained for Pax7 (green) five days after seeding on dECM-treated or untreated plates. Nuclei are stained blue. (**D**) Bar graphs show the total cell count per field, and (**E**) the percentage of Pax7^+^ satellite cells. (**F**) Analysis of TAs in athymic nude Foxn1nu mice transplanted with dECM-treated or untreated human satellite cells. Immunofluorescence images show myofibers positive for human dystrophin (red) 28 days after transplantation. Nuclei are stained blue. Bar graphs indicate the number of dystrophin-positive myofibers per cluster. (**G**) Mass spectrometry analysis of dECM and protein classification reveals six main categories related to skeletal muscle and stem cells: structure and organization, development, regeneration, proliferation, differentiation, and maintenance. (**H**) Immunofluorescence images of Pax7^+^ satellite cells five days after treatment with laminin, collagen IV, nidogen, and perlecan alone or in combination. Nuclei are stained blue. (**I**) Bar graphs show the percentage of Pax7^+^ satellite cells under different conditions. Scale bars: 100–150 μm. **p < 0.01, ***p < 0.001

Satellite cells gradually lose their ability to self-renew and maintain stemness when removed from their in vivo niche, creating a significant barrier to both long-term in vitro expansion and therapeutic potential [3, 22]. Conditions that support their maintenance are essential for providing a stable, functional source of human satellite cells suitable for transplantation. Our previous study showed that extracellular matrix derived from decellularized muscle (dECM) can promote proliferation and preserve murine satellite cells [23]. To determine whether a similar effect occurs in human satellite cells, sorted satellite cells were seeded onto dECM-coated or untreated plates (Fig. 2A). By day 5, dECM cultures showed more than a twofold increase in cell number compared to untreated controls (Fig. 2B, D), along with a significantly higher proportion of Pax7^+^ satellite cells (92.40% vs. 87.35%, Fig. 2C, E), indicating that dECM not only enhances proliferation but also helps prevent the loss of satellite cell identity in vitro. Pax7 expression was also monitored on day 10, and dECM-treated samples maintained higher levels than those without dECM (Fig. S2A, B). However, the overall Pax7 percentage decreased as cells approached confluence and began to fuse. We then assessed whether the supportive role of dECM extends to the in vivo myogenic activities of human satellite cells. After 5 days of culture on dECM substrates or untreated plates, human satellite cells were sorted and transplanted into pre-injured tibialis anterior (TA) muscles of athymic nude Foxn1nu mice (Fig. 2F). Four weeks after transplantation, TA muscles were harvested and prepared for analysis. Immunofluorescence images showed a significantly higher number of myofibers expressing human dystrophin in TA muscles injected with dECM-treated satellite cells than in those injected with untreated cells (Fig. 2F). Dystrophin⁺ myofiber formation reflects the combined effects of engraftment, survival, and fusion, and serves as a valuable indicator of functional regeneration. We also evaluated myofiber function by measuring ATPase levels in TA muscles transplanted with dECM-treated satellite cells. ATPase content in muscle fibers reflects the amount of ATP available for muscle contraction. This measurement is essential for understanding muscle performance, energy metabolism, and fatigue. We found a lower percentage of slow-twitch type I fibers in dECM samples, indicating a rapid repair process (Fig. S2C, D). In addition, we monitored the contribution of donor human satellite cells to the satellite cell pool by measuring co-expression of human lamin A/C and Pax7. TA muscles transplanted with dECM-treated satellite cells showed increased donor satellite cell repopulation compared with untreated samples (Fig. S2E, F). These findings suggest that dECM priming enhances the self-renewal and regenerative capacity of human satellite cells, thereby increasing their regenerative potential. Overall, mimicking niche-like extracellular matrix signals could be an effective strategy to overcome the inherent limitations of the number of human satellite cells for therapeutic applications.

### Nidogen and perlecan, ECM derivatives, support human satellite cells

Because dECM supports human satellite cells and enhances their in vivo engraftment potential, we analyzed the dECM proteome by liquid chromatography-tandem mass spectrometry (LC–MS). Gene Ontology (GO) analysis revealed that 189 proteins are associated with skeletal muscle and stem cell biological processes (Fig. 2G and Supplemental Table), including components of the satellite niche. Building on our finding that dECM promotes satellite cell function, we examined key basement membrane components of the satellite cell niche, such as laminin, collagen type IV, nidogen, and perlecan [24, 25], which were high-confidence LC–MS hits. We tested how these proteins influence human satellite cell growth by culturing the cells on surfaces coated with either a single protein or a combination of proteins. On day 5, we measured the percentage of Pax7^+^ cells. Each protein alone increased human satellite cell retention compared with untreated controls, and the combination of all four proteins yielded the highest percentage of Pax7^+^ satellite cells (Fig. 2H, I). To characterize satellite cell interactions with the substrate, we investigated β1-integrin signaling. β1-integrin, particularly the α2/β1 heterodimer, serves as a key receptor for perlecan, binding primarily to perlecan's C-terminal domain V (endorepellin) and thereby modulating cell adhesion and tissue repair [26, 27]. To validate satellite cell-substrate interactions, we performed an antibody-blocking assay, demonstrating that blocking β1-integrin significantly reduced satellite cell expansion compared with untreated samples (Fig. S2H, I). All these results highlight the synergistic role of these components in maintaining human satellite cells, consistent with their functions in the native muscle niche [28, 29].

## Discussion

The quality and quantity of human satellite cells influence both basic research and therapeutic applications. Cell surface markers such as CD29/CD56 or CD29/CD56/CXCR4 are commonly used to isolate human muscle stem cells [30–33]. However, CD56 is also expressed on proliferating myoblasts and non-myogenic cells [34–36], and CXCR4 levels can vary between donors, reducing consistency and purity during isolation (Cerletti, unpublished). Other markers, such as CD82 and CD318, have been explored to improve purification [37, 38]; however, they are present across many cell types [39–41]. Therefore, combining negative selection targeting CD31, CD34, CD45, and Mac1 to remove endothelial, stromal, and hematopoietic cells with positive selection for CD29 and CDH15, using satellite cell adhesion molecules, yields a more effective method for isolating satellite cells.

Our sorted CD31⁻CD34⁻CD45⁻Mac1⁻CD29⁺CDH15⁺ population contains a high proportion of Pax7^+^ cells and exhibits robust myogenic potential both in vitro and in vivo. CD29 regulates heterophilic interactions that anchor satellite cells to the basal lamina [18]. Additionally, CDH15 mediates homophilic adhesion between satellite cells and nearby myofibers, thereby stabilizing their position within the niche and maintaining quiescence [20]. This mechanistic link indicates that CDH15 is not only a structural surface marker but also a functional indicator of the stem-like state. Therefore, combining CDH15 with CD29 enriches for a lineage-restricted subset with high regenerative potential.

RNA probe-based labeling technologies have previously been used to observe gene expression in live cells, including neural stem cells and mesenchymal stromal cells [42–45]. However, to our knowledge, this is the first study applying this technology to detect and isolate Pax7^+^ human satellite cells, offering a new method for satellite cell purification. Here, we used a Pax7-targeting mRNA probe to fluorescently label live cells, enabling direct identification and subsequent isolation of human satellite cells. Compared with traditional surface marker-based sorting methods, the mRNA probe approach allows simultaneous visualization and tracking of Pax7^+^ cells' transcriptional activity during culture, supporting long-term studies of cell fate and behavior. Although labeling efficiency may vary with probe uptake, target transcript levels, and culture conditions [45, 46], the nanoparticle-based live Pax7 mRNA detection system is thoroughly validated using uptake and scrambled probe controls, as well as orthogonal validation. The results indicate that Pax7 mRNA probe-based labeling is a promising, complementary approach for the dynamic identification of human satellite cells within diverse myofiber-associated cell populations.

Additionally, we demonstrate that human satellite cells can remain undifferentiated and functional when cultured on muscle-derived extracellular matrix (dECM) or its derivatives. Mass spectrometry analysis of the dECM protein content highlights key basement membrane and satellite niche proteins—collagen IV, laminin, nidogen, and perlecan—all of which support satellite cell maintenance, with their combined presence having the most significant effect. Previous research has mainly focused on laminin, which promotes satellite cell self-renewal [47, 48]. These findings emphasize that, when combined, these proteins' roles underscore the importance of synergistic interactions among ECM components rather than the effects of individual molecules. In fact, the specific molecular mechanisms behind these cooperative effects remain poorly understood. These advancements could enhance the production, storage, and overall translational application of muscle precursors.

## Conclusions

In conclusion, we introduce two complementary strategies for efficiently isolating human satellite cells: a refined cell surface marker-based sorting method and a gold nanoparticle-based live Pax7 mRNA detection system. When combined with dECM-coated culture conditions, these techniques yield a stable, scalable source of functional human satellite cells. Together, these advances could address key challenges in obtaining consistent, high-quality human muscle stem cells, providing a solid foundation for future applications in personalized regenerative medicine, disease modeling, and the development of cell-based therapies for neuromuscular degeneration and traumatic muscle injury.

## Supplementary Information

Supplementary Material 1.

## Data Availability

No datasets were generated or analysed during the current study.
