# Supplementary material for: Isolation of human satellite cells using CD29/CDH15 and a nanoparticle-based live Pax7 mRNA detection system, and their expansion with extracellular matrix and its derivatives
Source: Stem Cell Res Ther. 2026 Jul 7;17:345. doi: 10.1186/s13287-026-05134-x (PMC13637147; doi:10.1186/s13287-026-05134-x)
Supplement: Supplementary file 1 [file 13287_2026_5134_MOESM1_ESM.pdf]

Supplemental Information

Isolation of human satellite cells using CD29/CDH15 and a nanoparticle-based live Pax7 mRNA detection system, and their expansion with extracellular matrix and its derivatives

Shudong Zhao[1,2,3], Shubhankar Sood[1,2,3], Ruixiang Yu[1,2,3], Luis Arvizu Gutierrez[1,2,3], Takhmina Ayupova[1,2,3], Sara Taiyari[1,2,3], Jonathan Miles[4], Stephen Hamilton[1,5], Massimiliano Cerletti[1,2,3]*

[1]Tissue Repair and Regeneration Centre, Division of Surgery & Interventional Science, University College London, Royal Free Campus, London, NW3 2PF, UK

[2]Centre for Surgical Innovation, Organ Repair and Transplantation (CSIORT), University College London, Royal Free Campus, London, NW3 2PF, UK

[3]Centre for Bioengineering and Surgical Technology (BEST), University College London, Brockley Hill, Stanmore, HA7 4AP, UK

[4]The Royal National Orthopaedic Hospital, Orthopaedic Surgery, Brockley Hill, Stanmore, HA7 4AP, UK

[5]The Royal Free Hospital, Breast and Plastic Surgery, London, NW3 2QG, UK

*Corresponding author. Email: m.cerletti@ucl.ac.uk (M.C.)

Supporting Table. List of gene ontology (GO) categories related to skeletal muscle and stem cell biological processes

Protein Symbol	Protein Name	Protein Symbol	Protein Name
**BIOLOGICAL PROCESS OF SKELETAL MUSCLE**			
**Category:** Extracellular Structure Organization			
ACAN	Aggrecan core protein	ELN	Elastin
ACSL3	Fatty acid CoA ligase Acsl3	EMILIN1	EMILIN-1
ADAMTSL4	ADAMTS-like protein 4	FBLN5	Fibulin-5
AEBP1	Adipocyte enhancer-binding protein 1	FECH	Ferrochelatase, mitochondrial
		FN1	Fibronectin
ANXA2	Annexin A2	HSD17B12	Very-long-chain 3-oxoacyl-CoA reductase
APOA4	Apolipoprotein A-IV		
APOE	Apolipoprotein E	HSPG2	Basement membrane-specific heparan sulfate proteoglycan core protein
ARF1	ADP-ribosylation factor 1		
CAV1	Caveolin-1		
CAV2	Caveolin-2	ITGB1	Integrin beta-1
CD36	Platelet glycoprotein 4	ITIH1	Inter-alpha-trypsin inhibitor heavy chain H1
CHADL	Chondroadherin-like protein		
		LAMB1	Laminin subunit beta-1
COL1A1	Collagen alpha-1(I) chain	LAMB2	Laminin subunit beta-2
COL1A2	Collagen alpha-2(I) chain	LAMC1	Laminin subunit gamma-1
COL2A1	Collagen alpha-1(II) chain	LOX	Protein-lysine 6-oxidase
COL3A1	Collagen alpha-1(III) chain	LOXL2	Lysyl oxidase homolog 2
COL4A1	Collagen alpha-1(IV) chain	LUM	Lumican
COL4A2	Collagen alpha-2(IV) chain	MIA3	Transport and Golgi organization protein 1 homolog
COL5A1	Collagen alpha-1(V) chain		
COL5A2	Collagen alpha-2(V) chain	MYH11	Myosin-11
COL9A1	Collagen alpha-1(IX) chain	NID1	Nidogen-1
COL9A2	Collagen alpha-2(IX) chain	P4HA1	Prolyl 4-hydroxylase subunit alpha-1
COL10A1	Collagen alpha-1(X) chain		
COL11A1	Collagen alpha-1(XI) chain	PDCD6IP	Programmed cell death 6-interacting protein
COL11A2	Collagen alpha-2(XI) chain		
COL14A1	Collagen alpha-1(XIV)	PHLDB1	Pleckstrin homology-like domain

	chain		family B member 1
COL15A1	Collagen alpha-1(XV)	PLG	Plasminogen
	chain	POSTN	Periostin
COL16A1	Collagen alpha-1(XVI)	PRDX4	Peroxiredoxin-4
	chain	PXDN	Peroxidasin homolog
COL18A1	Collagen alpha-1(XVIII)	SERPINH1	Serpin H1
	chain	TGFBI	Transforming growth factor-beta-
COL28A1	Collagen alpha-1(XXVIII)		induced protein ig-h3
	chain	TMEM38B	Trimeric intracellular cation
DAG1	Dystroglycan 1		channel type B
DGAT1	Diacylglycerol O-	TSG101	Tumor susceptibility gene 101
	acyltransferase 1		protein
DNAJB6	DnaJ homolog subfamily B	VTN	Vitronectin
	member 6	VWA1	von Willebrand factor A domain-
DPT	Dermatopontin		containing protein 1

Category: Skeletal Muscle Tissue Development

ACTA1	Actin, alpha skeletal muscle	LMOD3	Leiomodin-3
ACTN3	Alpha-actinin-3	MAPK14	Mitogen-activated protein kinase
ANKRD2	Ankyrin repeat domain-		14
	containing protein 2	METTL21C	Protein-lysine methyltransferase
ASB2	Ankyrin repeat and SOCS		METTL21C
	box protein 2	MYH14	Myosin-14
BVES	Blood vessel epicardial	MYL3	Myosin light chain 3
	substance	MYL6	Myosin light polypeptide 6
CACNA1S	Voltage-dependent L-type	MYL6B	Myosin light chain 6B
	calcium channel subunit	MYLK2	Myosin light chain kinase 2,
	alpha-1S		skeletal/cardiac muscle
CASQ1	Calsequestrin-1	MYLPF	Myosin regulatory light chain 2,
CAV1	Caveolin-1		skeletal muscle isoform
CAV2	Caveolin-2	MYOM1	Myomesin-1
CDK5	Cyclin-dependent-like	MYORG	Myogenesis-regulating glycosidase
	kinase 5	MYOZ1	Myozenin-1
CFL2	Cofilin-2	MYOZ2	Myozenin-2
COPS2	COP9 signalosome	NRAS	GTPase NRas
	complex subunit 2	POPDC2	Popeye domain-containing protein
CTNNB1	Catenin beta-1		2
DCN	Decorin	RHOA	Transforming protein RhoA

DDX17	Probable ATP-dependent RNA helicase DDX17	RPS6KB1	Ribosomal protein S6 kinase beta-1
DDX5	Probable ATP-dependent RNA helicase DDX5	RYR1	Ryanodine receptor 1
DMD	Dystrophin	SMYD1	Histone-lysine N-methyltransferase Smyd1
EEF2	Elongation factor 2	STAC3	SH3 and cysteine-rich domain-containing protein 3
ELN	Elastin	SVIL	Supervillin
EMD	Emerin	UQCC2	Ubiquinol-cytochrome-c reductase complex assembly factor 2
FLNB	Filamin-B	USP2	Ubiquitin carboxyl-terminal hydrolase 2
GPC1	Glypican-1	VAMP5	Vesicle-associated membrane protein 5
GPX1	Glutathione peroxidase 1	YBX3	Y-box-binding protein 3
HOMER1	Homer protein homolog 1		
KLHL40	Kelch-like protein 40		
KLHL41	Kelch-like protein 41		
LEMD2	LEM domain-containing protein 2		

Category: Skeletal Muscle Regeneration

ANXA1	Annexin A1	GPX1	Glutathione peroxidase 1
CD81	CD81 antigen	MAPK14	Mitogen-activated protein kinase 14
DAG1	Dystroglycan 1	MYOZ1	Myozenin-1
DMD	Dystrophin	PKM	Pyruvate kinase PKM
DYSF	Dysferlin	SGCA	Alpha-sarcoglycan
ENO3	Beta-enolase		

BIOLOGICAL PROCESS OF SKELETAL MUSCLE STEM CELLS

Category: Stem Cell Proliferation

ACSL6	Long-chain-fatty-acid--CoA ligase 6	LAMA2	Laminin subunit alpha-2
ARIH2	E3 ubiquitin-protein ligase ARIH2	MGP	Matrix Gla protein
CTNNA1	Catenin alpha-1	NDE1	Nuclear distribution protein nudE homolog 1
CTNNB1	Catenin beta-1	NES	Nestin
EEF1G	Elongation factor 1-gamma	PAFAH1B1	Platelet-activating factor acetylhydrolase IB subunit beta
EML1	Echinoderm microtubule-associated protein-like 1	RAB10	Ras-related protein Rab-10
HNRNPU		VCAN	Versican core protein
		YAP1	Transcriptional coactivator YAP1

ITGB1	Heterogeneous nuclear ribonucleoprotein U<br>Integrin beta-1		

Category: Stem Cell Differentiation

ANXA6	Annexin A6	MSI2	RNA-binding protein Musashi homolog 2
CFL1	Cofilin-1		
CSRP3	Cysteine and glycine-rich protein 3	OCIAD1	OCIA domain-containing protein 1
		PDCD6	Programmed cell death protein 6
FN1	Fibronectin	PEF1	Peflin
GSK3B	Glycogen synthase kinase-3 beta	PSMD11	26S proteasome non-ATPase regulatory subunit 11
HES1	Transcription factor HES-1	RAB10	Ras-related protein Rab-10
HNRNPU	Heterogeneous nuclear ribonucleoprotein U	STAT3	Signal transducer and activator of transcription 3
HSPA9	Stress-70 protein, mitochondrial	TAPT1	Transmembrane anterior posterior transformation protein 1
LAMA5	Laminin subunit alpha-5	THOC2	THO complex subunit 2
		YAP1	Transcriptional coactivator YAP1

Category: Stem Cell Maintenance

CDC73	Parafibromin	FMOD	Fibromodulin
CDH13	Cadherin-13	HES1	Transcription factor HES-1
COL5A1	Collagen alpha-1(V) chain	HOOK3	Protein Hook homolog 3
COL5A2	Collagen alpha-2(V) chain	ITGA6	Integrin alpha-6
COL6A1	Collagen alpha-1(VI) chain	ITGB1	Integrin beta-1
COL6A2	Collagen alpha-2(VI) chain	LAMA2	Laminin subunit alpha-2
COL6A5	Collagen alpha-5(VI) chain	LOXL2	Lysyl oxidase homolog 2
COL6A6	Collagen alpha-6(VI) chain	PCM1	Pericentriolar material 1 protein
CREBBP	Histone lysine acetyltransferase CREBBP	PEF1	Peflin
		PELO	Protein pelota homolog
CSRP3	Cysteine and glycine-rich protein 3	PSMD11	26S proteasome non-ATPase regulatory subunit 11
CTNNB1	Catenin beta-1	STAT3	Signal transducer and activator of transcription 3
DCN	Decorin		
EEF1G	Elongation factor 1-gamma	YAP1	Transcriptional coactivator YAP1

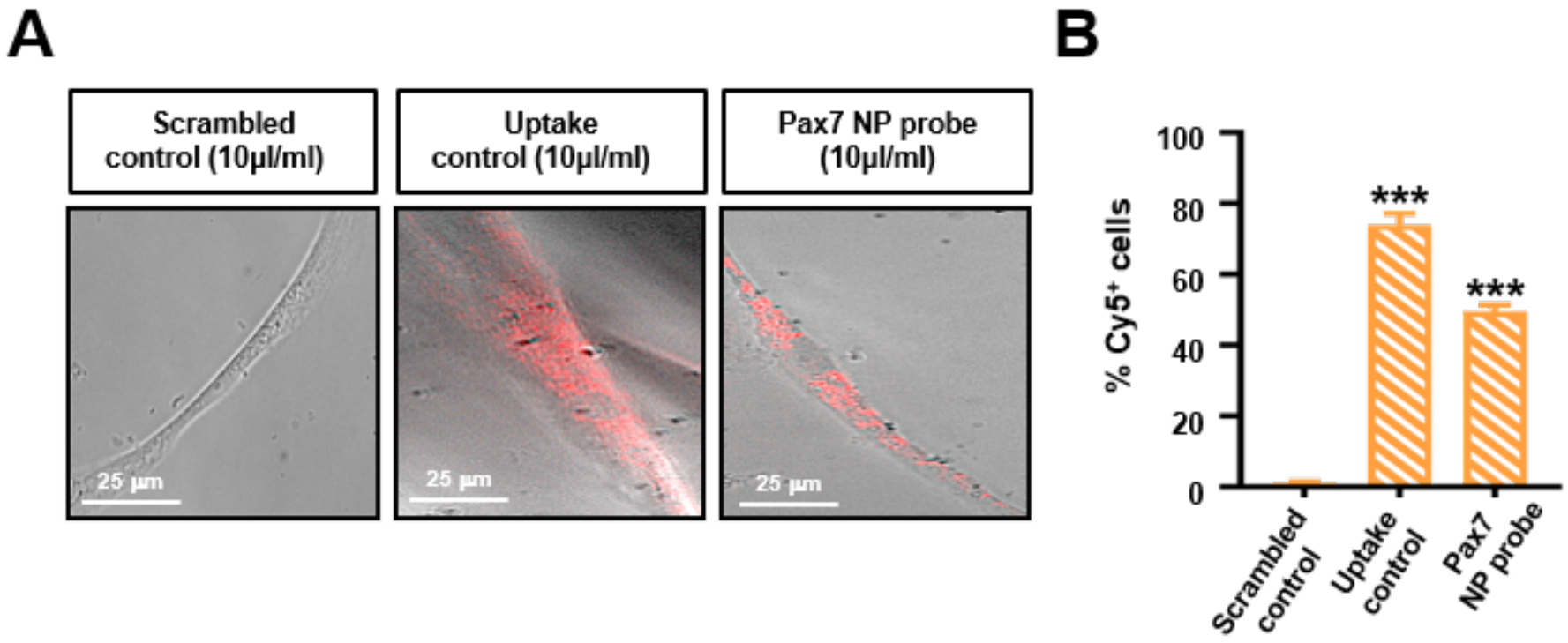

Figure S1. Validation of the nanoparticle-based Pax7 mRNA probe system. (**A-B**) Representative live images of $CD31^{-}CD34^{-}CD45^{-}Mac1^{-}$ cells incubated with the scrambled control, the uptake control, or the Pax7 NP probe at 10 µL/mL for 24 h. Bar graphs show the percentage of $Cy5^{+}$ cells. Scale bars: 25 µm. ***$p<0.001$.

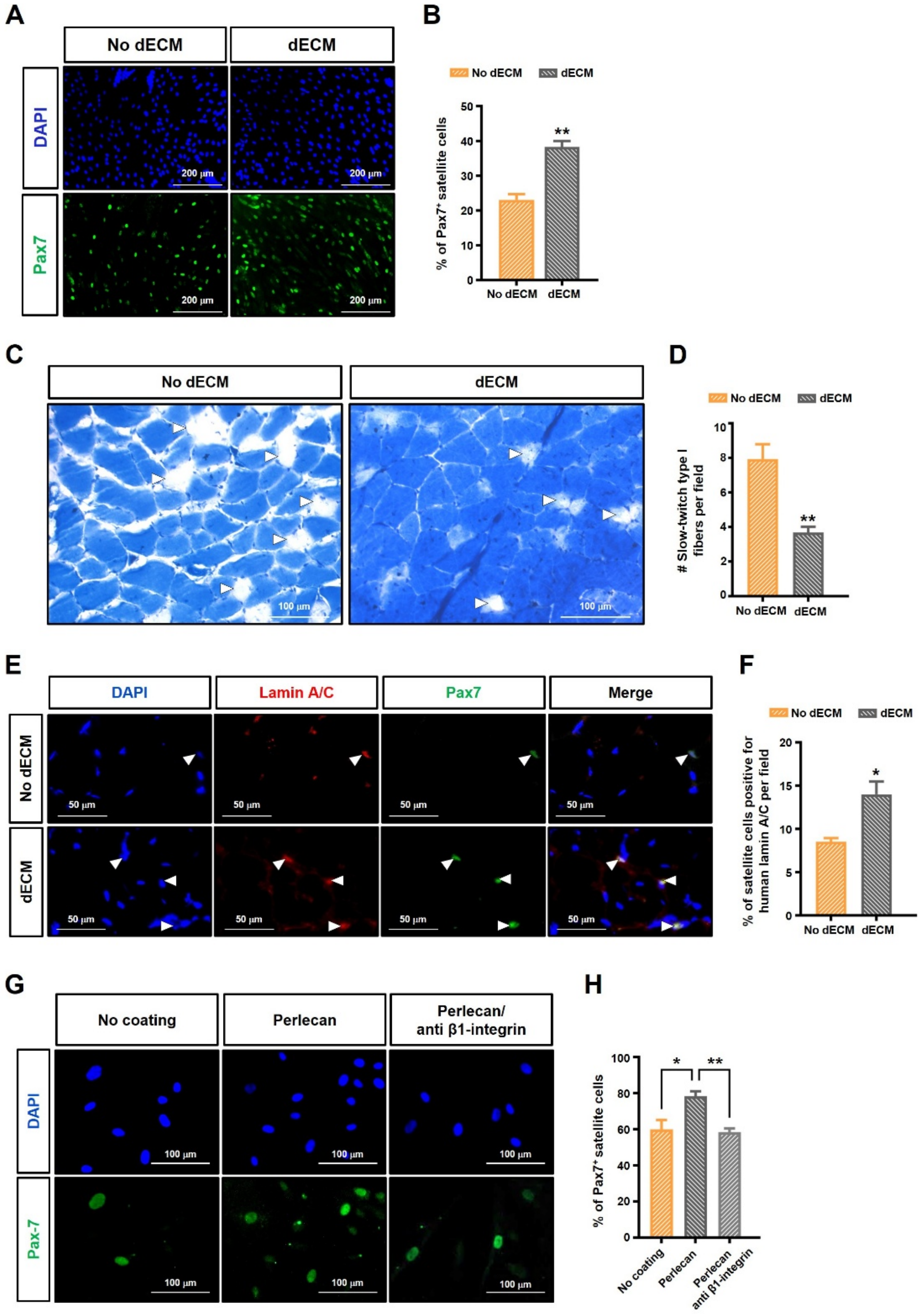

Figure S2. Additional characterization of human satellite cells treated with dECM and its derivatives *in vitro* and *in vivo*. (A) Immunofluorescence images of satellite cells stained for Pax7 (green) 10 days after seeding onto dECM-treated or untreated substrates confirm the difference observed at day 5 between the two experimental groups. Nuclei are stained blue. (**B**) Bar graphs show the percentage of Pax7$^+$ satellite cells. **(C)** Transplantation of dECM-treated human satellite cells into the TA muscle shows donor contribution, as evidenced by increased myofiber ATPase activity. ATPase content in muscle fibers reflects the amount of ATP available for muscle contraction. This is essential for understanding muscle performance, energy metabolism, and fatigue. TA muscles transplanted with dECM-treated satellite cells exhibited a lower percentage

of slow-twitch type I myofibers (white/light blue fibers, indicating low ATPase activity) and a predominance of fast-twitch type II myofibers (dark blue fibers, indicating high ATPase activity), compared with TA muscles transplanted with untreated satellite cells. **(D)** Bar graphs indicate the number of slow-twitch type I myofibers. **(E)** Donor contribution was also assessed by detecting $Pax7^+$ donor-derived cells in the satellite cell niche after transplantation. Analysis of Pax7 and human lamin A/C co-expression shows a higher percentage of $Pax7^+$/human lamin A/C^+ double-positive cells in TA muscles transplanted with dECM-treated satellite cells compared with those transplanted with untreated satellite cells. (**F**) Bar graphs show the percentage of double-positive cells. **(G)** Perlecan supports human satellite cell maintenance through interaction with β1-integrin. Immunofluorescence images of human satellite cells stained for Pax7 (green) five days after seeding on untreated substrates, perlecan-treated substrates, and perlecan-treated substrates with a β1-integrin blocking antibody. Nuclei are stained blue. (**H**) Bar graphs show the percentage of $Pax7^+$ satellite cells. Scale bars: 50-200 μm. $^*p<0.05$, $^{**}p<0.01$.
